# Supplementary material for: Melioidosis Knowledge Awareness in Three Distinct Groups in the Tropical Northern Territory of Australia
Source: Trop Med Infect Dis. 2024 Mar 28;9(4):71. doi: 10.3390/tropicalmed9040071 (PMC11054246; doi:10.3390/tropicalmed9040071)
Supplement: Supplementary file 1 [file tropicalmed-09-00071-s001.zip › tropicalmed-2926626-supplementary.pdf]

Tropical Medicine and Infectious Disease

MDPI

Supplementary Materials:

Melioidosis Knowledge Awareness in Three Distinct Groups in the Tropical Northern Territory of Australia

Madusha P. Weeratunga [1,2,3], Mark Mayo [1] and Mirjam Kaestli [1] and Bart J. Currie [1,2,3,*]

[1] Menzies School of Health Research, Charles Darwin University, Darwin, NT, 0811 Australia; madusha.weeratunga@nt.gov.au (M.P.W.); mark.mayo@menzies.edu.au (M.M.); mirjam.kaestli@menzies.edu.au (M.K.)
[2] Infectious Diseases Department, Royal Darwin Hospital, Darwin, NT, 0810 Australia
[3] Northern Territory Medical Program, Flinders and Charles Darwin University, Darwin, NT, 0810 Australia
* Correspondence: bart.currie@menzies.edu.au

QUESTIONNAIRE

Thank you for doing this short survey – it will take less than 5 minutes. We will use the survey to help you and your community from getting sick

General Information

1. Gender : ☐ Male ☐ Female ☐ Other
2. Age: _______ years
3. Number of years lived in the Northern Territory: ______
4. Highest level of education:
   - ☐ No schooling completed
   - ☐ Primary school (Grade 6 or equivalent)
   - ☐ High School (Grade 9 or equivalent)
   - ☐ College (Grade 12 or equivalent)
   - ☐ Apprenticeship or equivalent
   - ☐ University Bachelor's degree or equivalent
   - ☐ University degree higher than bachelor's degree or equivalent
5. Region /Post code: ______________

Have you ever heard of melioidosis?

☐ Yes ☐ No

Do you think these behaviours increase your chance of getting melioidosis?

1. Gardening in wet season	☐ Yes	☐ No	☐ Don't know
2. Hunting out bush during wet season	☐ Yes	☐ No	☐ Don't know
3. Playing football on community oval in wet season	☐ Yes	☐ No	☐ Don't know
4. Drinking sugary soft drinks	☐ Yes	☐ No	☐ Don't know
5. Drinking tap water	☐ Yes	☐ No	☐ Don't know
6. Smoking cigarettes	☐ Yes	☐ No	☐ Don't know
7. Drinking alcohol	☐ Yes	☐ No	☐ Don't know
8. Walking without shoes	☐ Yes	☐ No	☐ Don't know
9. Sharing needles for injections	☐ Yes	☐ No	☐ Don't know

Do you think these actions protect you from getting melioidosis?

1. Wearing boots and rubber gloves when working outdoors	☐ Yes	☐ No	☐ Don't know
2. Wearing shoes when walking outside	☐ Yes	☐ No	☐ Don't know
3. Covering wounds with band-aids	☐ Yes	☐ No	☐ Don't know
4. Staying indoors during storms	☐ Yes	☐ No	☐ Don't know
5. Drinking less alcohol	☐ Yes	☐ No	☐ Don't know
6. Eating lots of fruit and vegetables	☐ Yes	☐ No	☐ Don't know
7. Wearing a mask when using a high-pressure hose	☐ Yes	☐ No	☐ Don't know

Project: Top End Awareness of Melioidosis Project
(HREC 2021-4058)

How have you heard about melioidosis?

- ☐ I don't know what this is
- ☐ From my doctor or healthcare worker
- ☐ From a family member or friend
- ☐ On TV
- ☐ On Radio
- ☐ Newspaper
- ☐ At the clinic or hospital
- ☐ On a poster
- ☐ I can't remember
- ☐ Other, please tell us how ____

Where did you go to find more information about melioidosis?

- ☐ I didn't look for any information because it is not important to me
- ☐ I didn't know where to find any information
- ☐ I asked my doctor or healthcare worker
- ☐ I asked my family and friends
- ☐ I searched on the internet
- ☐ I read a pamphlet in the clinic or hospital
- ☐ I can't remember
- ☐ Other, please tell us how ____

What do you think is the best way to help people in your community avoid getting melioidosis? Tick all that apply

- ☐ Doctors and health care workers giving advice
- ☐ TV advertising
- ☐ Radio advertising
- ☐ On the Internet
- ☐ Posters around the community
- ☐ At schools so that my kids hear about it
- ☐ Other, please tell us how ____

Do you know the NT government website that has a melioidosis (also known as melioid) factsheet?

☐Yes
☐No

menzies school of health research
discovery for a healthy tomorrow
PO Box 41096, CASUARINA NT 0811 | John Mathews Building (Building 58), Royal Darwin Hospital Campus, Rocklands Drive, CASUARINA NT 0810 Phone: 08 8922 8196 | Facsimile: 08 8927 5187 | Web: www.menzies.edu.au | Email: info@menzies.edu.au

Figure S1. The questionnaire used in the study.

Table S1. Summary of the remaining survey questions.

How have you heard about melioidosis?						
**Variable**	**Group 1**		**Group 2**		**Group 3**	
	**Num-ber**	**Percentage**	**Num-ber**	**Percentage**	**Num-ber**	**Percentage**
I don't know what this is	5	0.04	16	0.32	1	0.04
From a family member or friend	48	0.38	14	0.28	3	0.11
On radio	14	0.11	6	0.12	1	0.04
At the clinic or hospital	36	0.29	8	0.16	8	0.29
I can't remember	11	0.09	3	0.06	1	0.04
From my doctor or healthcare worker	13	0.10	8	0.16	5	0.18
On TV	20	0.16	7	0.14	1	0.04
Newspaper	16	0.13	1	0.02	2	0.07
On a poster	24	0.19	4	0.08	4	0.14
Other	30	0.24	10	0.2	10	0.36
**Where did you go to find more information about melioidosis?**						
**Variable**	**Group 1**		**Group 2**		**Group 3**	
	**Num-ber**	**Percentage**	**Num-ber**	**Percentage**	**Num-ber**	**Percentage**
I didn't look for any information because it is not important to me	17	0.14	13	0.26	1	0.04
I didn't know where to find any information	6	0.05	7	0.14	6	0.21
I asked my doctor or healthcare worker	15	0.12	17	0.34	12	0.43

I asked my family and friends	10	0.08	7	0.14	4	0.14
I searched on the Internet	84	0.67	11	0.22	6	0.21
I read a pamphlet in the clinic or hospital	11	0.09	9	0.18	2	0.07
I can't remember	7	0.06	1	0.02	2	0.07
Others	14	0.11	0	0	2	0.07

What do you think is the best way to help people in your community avoid getting melioidosis?

Variable	Group 1		Group 2		Group 3	
	Num-ber	Percentage	Num-ber	Percentage	Num-ber	Percentage
Doctors and health care workers giving advice	87	0.70	35	0.7	21	0.75
Radio advertising	61	0.49	30	0.6	11	0.39
Posters	74	0.59	29	0.58	8	0.29
TV advertising	78	0.62	16	0.32	15	0.54
On the Internet	66	0.53	18	0.36	8	0.29
At schools so that my kids hear about it	85	0.68	31	0.62	13	0.46
Other	15	0.12	8	0.16	4	0.14
